# Participation of the* HIM1* gene of yeast *Saccharomyces cerevisiae* in the error-free branch of post-replicative repair and role Polη in *him1*-dependent mutagenesis

**DOI:** 10.1007/s00294-020-01115-6

**Published:** 2020-10-31

**Authors:** E. A. Alekseeva, T. A. Evstyukhina, V. T. Peshekhonov, V. G. Korolev

**Affiliations:** 1 Division of Molecular and Radiation Biophysics, Laboratory of Eukaryote Genetics, Petersburg Nuclear Physics Institute named by B.P. Konstantinov of National Research Centre, Kurchatov Institute, mkr. Orlova Roscha 1, 188300 Gatchina, Russia; 2 Division of Molecular and Radiation Biophysics, Laboratory of Eukaryote Genetics, 1)Petersburg Nuclear Physics Institute named by B.P. Konstantinov of National Research Centre «Kurchatov Institute», 2) Kurchatov Genome Center – PNPI, mkr. Orlova Roscha 1, Leningrad District, 188300 Gatchina, Russia

**Keywords:** *HIM1* gene, Yeast, Mutagenesis, DNA damage, Post-replication repair

## Abstract

In eukaryotes, DNA damage tolerance (DDT) is determined by two repair pathways, homologous repair recombination (HRR) and a pathway controlled by the RAD6-epistatic group of genes. Monoubiquitylation of PCNA mediates an error-prone pathway, whereas polyubiquitylation stimulates an error-free pathway. The error-free pathway involves components of recombination repair; however, the factors that act in this pathway remain largely unknown. Here, we report that the *HIM1* gene participates in error-free DDT. Notably, inactivation *RAD30* gene encoding Polη completely suppresses *him1*-dependent UV mutagenesis. Furthermore, data obtained show a significant role of Polη in *him1*-dependent mutagenesis, especially at non-bipyrimidine sites (NBP sites). We demonstrate that *him1* mutation significantly reduces the efficiency of the induction expression of *RNR* genes after UV irradiation. Besides, this paper presents evidence that significant increase in the dNTP levels suppress *him1*-dependent mutagenesis. Our findings show that Polη responsible for *him1*-dependent mutagenesis.

## Introduction

Growing cells need to replicate their DNA with high fidelity to avoid genome instability. During DNA replication, replication fork sometimes encounters a lesion in the DNA template, leading to polymerase stalling. To prevent a replication arrest, cells employ DNA damage bypass mechanisms that allow the complete replication of the genome in the presence of lesions (Friedberg 2005). These DNA damage tolerance processes contribute to survival after DNA damage and sometimes also actively promote the generation of mutations. DNA-damage tolerance was termed post-replication repair (PRR) due to the observation that the treatment of budding yeast cells with UV irradiation caused single-strand DNA gaps (Prakash 1981). The UV-induced pyrimidine dimers in the DNA were often retained following “repair”, indicating that PRR simply bypasses the damage (Ganesan 1974). Two distinct modes of DDT, error-prone and error-free DDT, operate in all eukaryotic organisms (Sale 2012). Error-prone DDT is mediated by translesion synthesis (TLS), and in error-free pathway, one newly synthesized strand serves as a replication template for the other blocked nascent strand (Branzei et al. 2008; Giannattasio et al. 2014). Cells defective in error-free DDT are characterized by dependency on TLS, showing higher levels of spontaneous mutagenesis and increased damage sensitivity following the inactivation of error-prone DDT components (Cejka et al. 2001). Mutations in the error-free pathway confer a greater sensitivity to DNA-damaging agents than mutations in the TLS pathway (Brusky et al. 2000).

In eukaryotes, two types of damage bypass are controlled by differential ubiquitylation of the proliferating cell nuclear antigen (PCNA), via components of the *RAD6* pathway (Ulrich 2009). PCNA is monoubiquitylated on a lysine residue, K164, by the pair Rad6–Rad18, which promotes the recruitment of DDT components capable of replicating damaged DNA in a TLS. Extension of this modification with a polyubiquitin chain by the Ubc13-Mms2 and the Rad5 promotes an error-free pathway called template switching (TS).

In recent years, a number of additional factors involved in the TS were reported. In addition to the enzymes promoting PCNA polyubiquitylation, these include the 9-1-1 checkpoint clamp and the Exo1 exonuclease, which contributes to Rad53-dependent activation by widening replication gaps, proteins mediating the strand invasion step (Rad52 and Shu complex), as well as the helicase Sgs1, implicated in the resolution of TS intermediates (Karras et al. 2013; Vanoli et al. 2010; Ball et al. 2009).

To reveal genes, which control the process of induced mutagenesis, we have developed a method of isolation of the yeast mutants affecting the pathway by the phenotype of enhanced induced mutagenesis. Six mutants have been isolated and designated *him* (*h*igh *i*nduced *m*utagenesis). The *him1Δ* mutation was induced by nitrosomethylurea and isolated by its feature to increase the frequencies of the nitrous acid-induced *ade2-42* reversions (Ivanov et al. 1987a, b). The *him1Δ* mutant displayed enhanced rates of reversion of the *ade2-42* allele, and forward mutations in the *ADE1* and *ADE2* genes induced by nitrous acid (NA) and UV light. The analysis of the genetic interaction of *him1Δ* mutation with mutations blocking three different repair pathways revealed that the gene, *HIM1*, participates in post-replication repair (the *RAD6* pathway) and recombinational repair (the *RAD52* pathway) (Kelberg et al. 2005). In this work, we have shown by genetic methods that the *HIM1* gene takes part in the error-free branch of post-replicative repair and *him1Δ* mutant recruitment of Polη in reparative DNA synthesis.

## Materials and methods

### Materials

A PCR-generated *natMX6* module and a PCR module containing the *URA4* gene from *Schizosaccharomyces pombe* were amplified from pFLA6A-natMX6 and pFLA6a-URA4 plasmids (Latypov) using *SML1*_DelL: 5′-TGTCTTATCTGCTCCTTTGTGATCTTACGGTCTCACTAACCTCTCTTCAACTGCTCAATAATTTCCCGCTGCTTCGTACGCTGCAGGTCG-3′; *SML1*_DelR: 5′-CGAGAATGACAACAATAGTAGGACGAGAGTCCCTGAAAAGAAGGGTATCTAAGAGAAGAAAAGAACAGAAGCATAGGCCACTAGTGGATC; *RAD30* DelL: 5′-ACTTGGAAGGAGTTGATTCAGCTTGGTTCCCCCAGTAAAGCATACGAGTCCTCCTTAGCATGTATCGCCCGCTTCGTACGCTGCAGGTCG-3′; *RAD30* DelR: 5′-CTTGTAAAAAATGATAAGATGTTTTTGGAAGATGTAACTTGTTTCTTCTGAGGTGTGGCAGTATGTTGTGGCATAGGCCACTAGTGGATC-3′; *MMS2*_del_L: 5′-TCGATGTCGTGGTGAAATTCTTATTCTGTATATGCAACGTAGAAGAAAGCAGCGTTTACACAAAAATGTCGCTTCGTACGCTGCAGGTCG-3′; *MMS2* del_R: 5′-TTGGAATGCTGCAAATACTGTTTAGGAAAAAGTAGATAACTAAAAGGTTTCTCCTTCCTTCGGTTGACGCGCATAGGCCACTAGTGGATC-3′ and *XRS2* del_L: ATATAAATGACAGCTTTTTATACATATAGACCCTTTGAAGAATATTCCAAACTAGAAAGGTTGATCAGAAGCTTCGTACGCTGCAGGTCG; *XRS2* del_R: 5′-TGGTTCTTTTATGTATTAGGCTACTATTTATTTAATAACTTCGCATCTATCAAAAGAAAAGACTGACTGTGCATAGGCCACTAGTGGATC deoxyoligonucleotides, respectively.

### Yeast strains and media

For culture growth and survival registration, the full medium was used (Zakharov et al. 1984). Alcohol containing medium was used in studies of the UV-induction of mutagenesis (Koval’tsova and Korolev 1996). *S. cerevisiae* strains used in this work are described in Table 1. The *xrs2Δ* (2ETA-3031) mutant were obtained from the previously described 11D-3031 (*MATα ade2Δ-248 leu2-3,112 ura3-160,188 trp1*) strain by gene replacement (Fedorova et al. 2004; Chernenkov et al. 2012a, b). The 11D-3031 strain was transformed with modules, and the transformants were selected on plates with YEPD containing 30 mg/l nourseothricin and on plates with selective media without uracil, respectively. The single mutants *him1Δ*(1-EAA-3031), *him1*(*1-EAA-3031*)*, sml1Δ*(6-DVF-3031)*, rad30 Δ*(4-EAA-3031), and *mms2Δ*(6-EAA-3031) and *xrs2Δ*(2-ETA-3031) were constructed from previously described 11D-3031, already referred to above. The double*, him1Δ rad30Δ*(*5-EAA-3031*)*, him1 sml1Δ*(7DVF-3031), *him1Δ rad30Δ*(5-EAA-3031), *him1Δ mms2* (7-EAA-3031) and *him1Δ xrs2Δ*(8-EAA-3031) mutants were also constructed from previously described 11D-3031. All mutants were PCR-verified.

**Table 1:** Yeast strains used in the work

Strain	Genotype
11D-3031	*MATα ade2Δ -248 ura3-160,188 leu2-3,112 trp1*
1-EAA-3031	*MATα ade2Δ-248 ura3-160,188 leu2-3,112 trp1 him1::URA4*
6- DVF-3031	*MATα ade2Δ-248 ura3-160,188 leu2-3,112 trp1 sml1::kanMX*
7- DVF-3031	*MATα ade2Δ-248 ura3-160,188 leu2-3,112 trp1 sml1::kanMXhim1:: URA4*
4-EAA-3031	*MATα ade2Δ-248 ura3-160,188 leu2-3,112 trp1rad30::kanMX*
5-EAA-3031	*MATα ade2Δ-248 ura3-160,188 leu2-3,112 trp1rad30::kanMX* *him1:: URA4*
6-EAA-3031	*MATα ade2Δ-248 ura3-160,188 leu2-3,112 trp1mms2::kanMX*
7-EAA-3031	*MATα ade2Δ-248 ura3-160,188 leu2-3,112 trp1mms2::kanMX him1::URA4*
2-ETA-3031	*MATα ade2Δ-248 ura3-160,188 leu2-3,112 trp1 xrs2::URA3*
8-EAA-3031	*MATα ade2Δ-248 ura3-160,188 leu2-3,112 trp1 xrs2::URA3* *him1:: URA4*

### Sensitivity against UV irradiation

Cell sensitivity against UV irradiation tests were performed on plates by growing an overnight culture of the respective strain in liquid YPD at 30 °C. Cells were washed and resuspended in water at a density of 1 × 10^7^ cell/ml. Cells were irradiated with a UV lamp BUV-30 (UV-C range). Aliquots were withdrawn at different times, diluted, and plated onto YPD plates to determine the number of survivors.

### UV mutagenesis assays

UV-induced mutagenesis was measured by registering mutations at five ADE loci (Roman 1956). Mutation tests were performed on plates by growing an overnight culture of the respective strain in liquid YPD at 30 °C. Cells were washed and resuspended in water at a density of 1 × 10^7^ cell/ml. Cells were irradiated with a UV lamp BUF-30. Aliquots were withdrawn at different times, diluted, and plated onto YPD plates to determine the number of survivors. To determine mutation frequency, aliquots without dilution were plated onto a medium YPD containing ethanol instead of glucose, the composition of which has been described earlier (Koval’tsova and Korolev 1996).

In total, five replicates of the experiment were performed; the mean values with 95% confidence intervals are given on the graphs and in the tables.

### *can*^*R*^* mutation spectra*

Yeast cell suspensions were irradiated with UV light at 84 J/m^2^. Then, cells were harvested by centrifugation and plated on selective medium with canavanine. Independent canavanine-resistant clones were transferred to fresh medium with canavanine. Genomic DNA was isolated from purified *can*^*R*^ colonies using a glass bead lysis procedure. A portion of *can*^*R*^ locus containing 800 bp was amplified and DNA sequence analysis of PCR-amplified genomic fragments was performed by “Beagle”, using the primers 5′-cacaacctctttcacgacg-3 and 5′-ggaaacccaacctaagaacc-3′.

### Real-time PCR

For conducting real-time PCR was used on a CFX96 RT-PCR Detection system (Bio-Rad, UK). The reactions were carried out in 25 µl volumes consisting of 10 µl 2.5-fold reaction mixture for RT-PCR in the presence SYBR Green I dye and Rox reference dye (Syntol, Russia), 14.1 µl water, 0.7 µl of cDNA and 0.1 (2 mM) respective primers (primers for gene *RNR3*: For*NR3* 5′-ACACCTTTCATGGTTTATAAG-3′ and Rev*RNR3* 5′-CGACGATTTCACAACATAA-3′; for gene *ACT1*: For*ACT1* 5′-GAAGGTCAAGATCATTGC-3′ and Rev*ACT1* 5′- GTTGGAAGGTAGTCAAAG-3′).

PCR cycling conditions were as follows: 1 cycle of 5 min at 95 °C, followed by 39 cycles of 15 s at 95 °C and 20 s at 52 °C. Melting curve analysis was 5 s incremental increases of 1 °C from 55 to 95 °C.

Control reactions with primer and template free reaction mixtures were included. Two biological and three technical replicates were performed for each sample. The results were processed using the CFX Manager program.

## Results

The response to DNA damage has been well characterized in *Saccharomyces cerevisiae*. It includes three groups of proteins involved in different types of DNA repair, termed the *RAD3, RAD52* and *RAD6* epistasis groups. UV response is mediated by the *RAD3* group. The *RAD52* epistasis group genes have been implicated in the response to DNA double strand breaks. Although *RAD3* and *RAD52* repair pathways are relatively well understood, much less is known about the *RAD6* mediated pathway. Mutations in the *REV3* gene, a member of the *RAD6* mutagenic repair pathway, are known to increase sensitivity to the lethal action of UV-light and to suppress mutagenesis. Our data demonstrate that *him1Δ* epistatically interacts with *rev3Δ*, since the level of UV-induced mutagenesis in double mutants was decreased to the level of mutagenesis in single *rev3Δ* mutant (Kelberg et al. 2005). Thus, we have suggested that *HIM1* gene participates in the PRR.

**Fig. 1:**
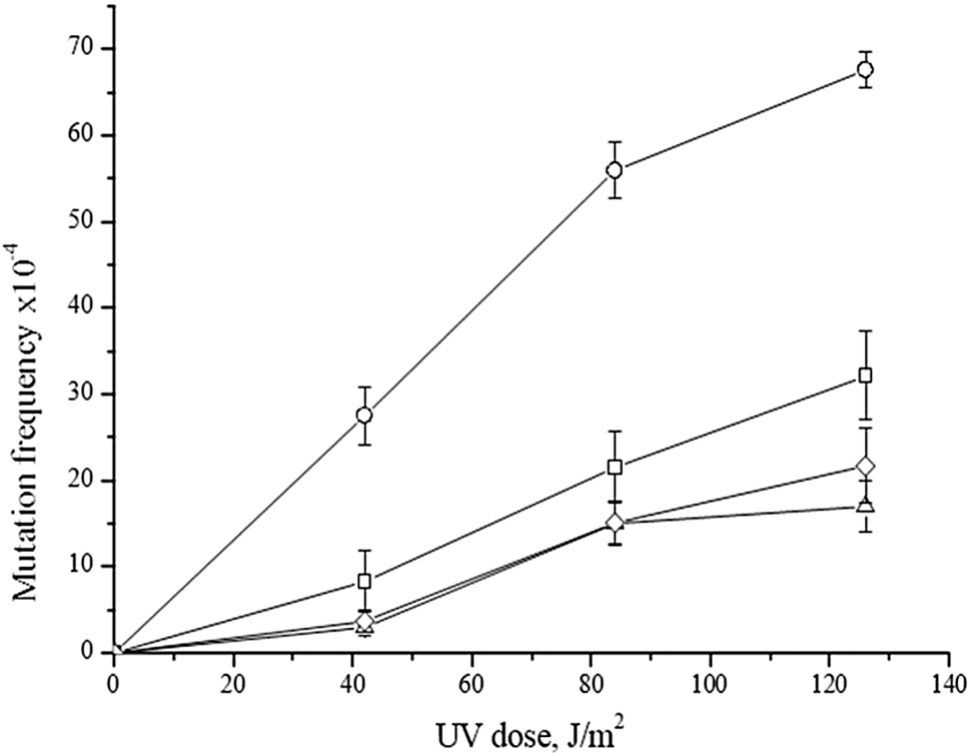
UV-induced mutagenesis in various mutant strains. Isogenic derivatives of strain 11D-3031, were UV-irradiated. The mutation frequencies were determined, as a ratio of the number of white colonies to the number of all colonies grown in a cup with complete medium. Mutation frequency is plotted on a linear scale graph. The mean ± SEM values obtained from four independent experiments are plotted. Mutant frequencies of the wild type strain (11D-3031) (open square); him1Δ (1-EAA-3031) (open cirle); mms2Δ (6-EAA-3031) (open triangle) and him1Δ mms2Δ (7-EAA-3031) (open diamond)

**Fig. 2:**
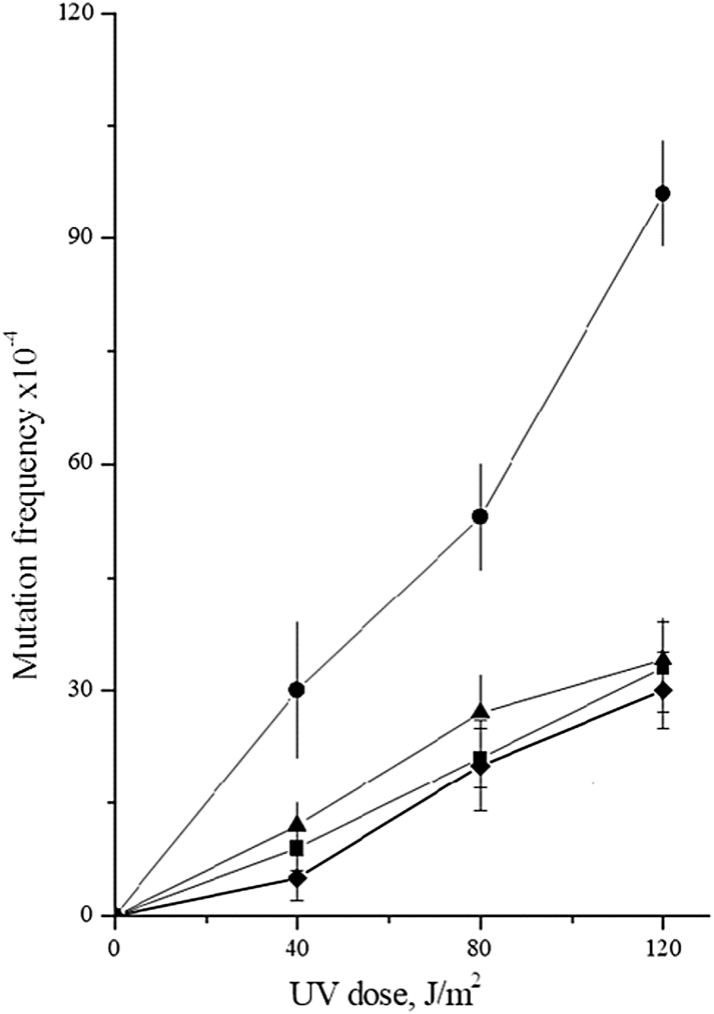
UV-induced mutagenesis in various mutant strains. Isogenic derivatives of strain 11D-3031, were UV-irradiated. The mutation frequencies were determined as described for Fig. 1. Mutant frequencies of the wild type strain (11D-3031) (closed square); *him1Δ* (1-EAA-3031) (closed circle); xrs2Δ (2-ETA-3031) (closed triangle) and *him1Δ* xrs2Δ (8-EAA-3031) (closed diamond)

Earlier, we have reported that spontaneous mitotic gene conversion in the *ADE2* locus in most heteroallelic combinations is increased (~ twofold) in *him1Δ* strains compared to the wild-type strain (Ivanov et al. 1989). These data suggest that *HIM1* gene may be involved in the control of the recombination events. In this regard, we can assume that the *HIM1* gene is involved in recombination (error-free) branch of PRR. The error-free post-replication repair involves the protein Rad5, with the assumed helicase activity (Johnson et al. 1992), and the protein Mms2, which acquires the ubiquitin-transferase activity when in complex with Ubc13p. Rad5-Mms2-Ubc13 provides PCNA polyubiquitylation, thus stimulating the recombination-like process associated with the helicase activity of Rad5p. *MMS2* gene, therefore, acts in the first stage of the error-free branch of PRR. To test the hypothesis, that *HIM1* gene may be involved in the control of the error-free branch of PRR, we deleted *MMS2* gene from *him1Δ* mutant and the wild-type strain. UV resistance of *mms2Δ him1Δ* double mutant did not differ from *mms2* single mutant (data not shown). As seen in Fig. 1, UV-induced mutagenesis in *mms2Δ* strain is markedly lower than in *him1Δ*, but is close to the level of mutagenesis in the wild-type strain. The error-prone branch of DDT has a seemingly limited ability to bypass large amounts of DNA damage, as supported by the work of Cejka et al (2001).Which showed that spontaneous damage in *rad5Δ* and *mms2Δ* mutants leads to an approximately fivefold increase in mutagenesis. With a sharp increase in the number of DNA damage caused by low UV doses (less than 10 J/m^2^), the level of induced mutagenesis increases by only 1.5 times (Broomfield and Xiao 2002). We work at high UV doses (at least 54 J/m^2^), at which, apparently, there is a further decrease in the effectiveness of the mutagenic effect of UV. This is also confirmed by the data in the work of Lemontt in which one of the first alleles of the *RAD5* gene was isolated according to the phenotype of a reduced level of UV-induced mutagenesis (Lemontt 1971). These data are consistent with our results that at high doses UV-induced mutagenesis in *mms2Δ* mutant is close to the wild-type strain. The double mutant exhibits mutagenesis the same as a single *mms2Δ* mutant. Therefore, *mms2Δ* is epistatic to *him1Δ* and *him1Δ* induced mutagenesis is Mms2 dependent. It was shown that ssDNA gaps behind stalled replication forks are due to Mre11-dependent degradation of nascent DNA. The engagement of these gaps in Rad51-dependent repair could prevent excessive nucleolytic degradation, sequestering the substrates once optimal Mre11-dependent resection is achieved (Hashimoto et al. 2010). This gap resection facilitates the invasion of the newly synthesized sister chromatid by the nascent DNA strand, thus initiating TS. Consequently, the inactivation of the MRX complex can disrupt the TS and, as a result, suppress the *him1*-dependent UV mutagenesis. To test this assumption, we examined potential epistatic relationships between mutations of *XRS2* and *HIM1* genes. *XRS2* encodes a subunit of the MRX complex (Rad50, Mre11, Xrs2). The UV resistance of *xrs2Δ him1Δ* double mutant did not differ from *xrs2* single mutant (data not shown). *xrs2Δ him1Δ* double mutant was as sensitive to UV-induced mutagenesis as *xrs2Δ* single mutant (Fig. 2). Taken together, these results strongly suggest that the *HIM1* gene is involved in the error-free branch of damage bypass.

In contrast to the key genes that control the error-free branch of the PRR (*RAD5, MMS2, MRE11*), the mutations of which have practically no effect on the frequency of UV-induced mutagenesis or even decrease it, the deletion of the *HIM1* gene leads to an increase in the yield of induced mutations (Kovaltzova 1973; Lemontt 1971). We were interested in understanding why there is a difference between him1 and the rest of the error free proteins, considering that all of them somehow participate in the error free? In our previous work, we showed that a mutation in the *HIM1* gene leads to destabilization of the D-loop (Alekseeva et al.^2018^). Based on these results, it can be assumed that during PRR at the stage of D-loop processing.

**Fig. 3:**
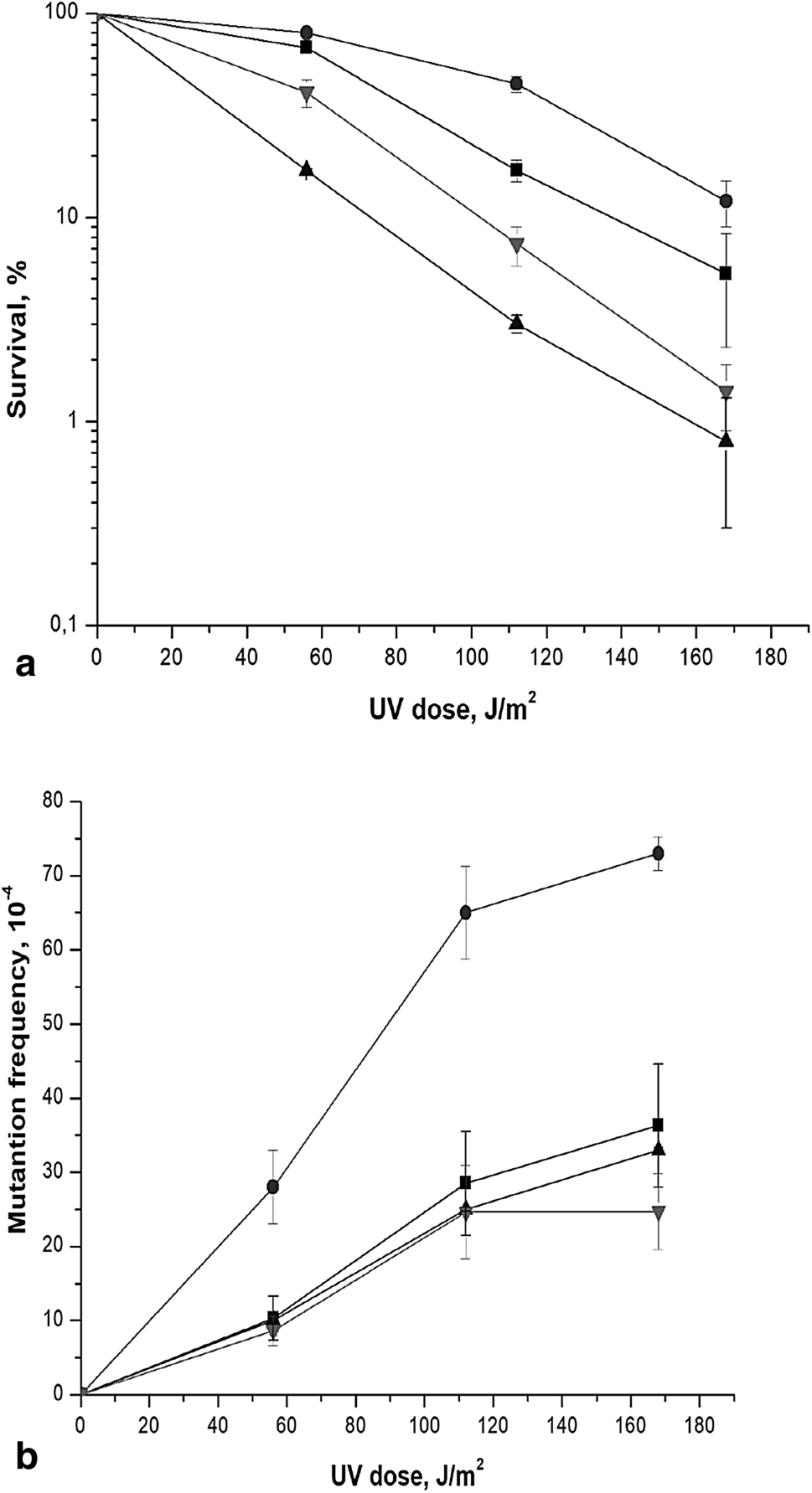
UV-induced sensativity (**a**) and mutagenesis (**b**) in various mutant strains. Isogenic derivatives of strain 11D-3031, were UV-irradiated. Aliquots were irradiated at the indicated dose, the viable titer was determined, and the percentage of survivals was calculated. The mutation frequencies were determined as described for Fig. 1. The wild type strain (11D-3031) (■); *himΔ* (1-EAA-3031) (•); *rad30Δ* (4-EAA-3031) (▲) and *him1Δ rad30Δ* (5-EAA-3031) (▼)

It has been shown that Polη and Polδ could extend the 3′ end in a D-loop (Li et al. 2009). Both polymerases were equally efficient in extending standard primed templates in vitro. Both Polη and Polδ require PCNA for processive DNA synthesis. We hypothesized that the cause of *him1*-mediated UV-induced mutagenesis is the replacement of Polδ with highly erroneous Polη during the DNA synthesis in gaps after the destruction of the D-loop. To test this assumption, we deleted *RAD30* gene in the wild-type strain and *him1Δ* mutant. The *rad30Δ* single mutant showed UV-induced mutagenesis as the wild-type strain (Fig. 3). The UV-induced mutagenesis in the double *him1Δ rad30Δ* mutant was the same as in the single *rad30Δ* mutant. Based on these results, it can be assumed that, in during PRR the Polη in *him1Δ* mutant carries out reparative synthesis in unfilled gaps.

Mutations that arise during TLS at bipyrimidine sites, mainly formed as a result of UV-induced damage. In contrast, mutations at non-bipyrimidine sites frequently occur as a result of gap filling with Polη. The *CAN1* gene sequence contains 77% and 23% non-bipyrimidine sites (NBP) (Kozmin et al 2003). It can be assumed that the frequency of UV-induced mutations localized in NBP sites in the *him1Δ* mutant will be higher than in the double *him1Δ rad3Δ* mutant. To get insight into the role of Polη in *him1*-dependent mutagenesis, mutation spectra were determined at the *CAN1* locus in *him1Δ* nd double *him1Δ rad30Δ* strains for UVC. We isolated 200 *can1* mutants following UV irradiation at dose 84 J/m^2^. The mutation frequencies were 31 × 10^–5^ and 10 × 10^–5^ in *him1Δ* and *him1Δ rad30Δ* mutants, respectively.

The UV-induced spectra generated in *him1* and *him1Δ rad30Δ* background are characterized by a predominance of single base substitutions (Table 2). The spectrum of mutations obtained by us in *him1Δ rad30Δ* mutant practically does not differ significantly from the mutation spectra in *rad30Δ* mutant obtained in the work (Kozmin et. al. 2003).

**Table 2:** DNA sequence changes in UV-induced *can1* mutants in *him1Δ* and *him1Δ rad30Δ* strains

Mutation type	*him1Δ*		*him1Δ rad30Δ*	
	*n* (%)	*f* × 10^–5^	*n* (%)	*f* × 10^–5^
Base substitutions	28 (74)	23	16 (70)	7
Frameshifts	4 (11)	3.4	5 (22)	2.2
Tandem double	4 (10)	3.1	1 (4)	0.4
Multiple	2 (5)	1.5	1 (4)	0.4

*f* mutation frequency

**Fig. 4:**
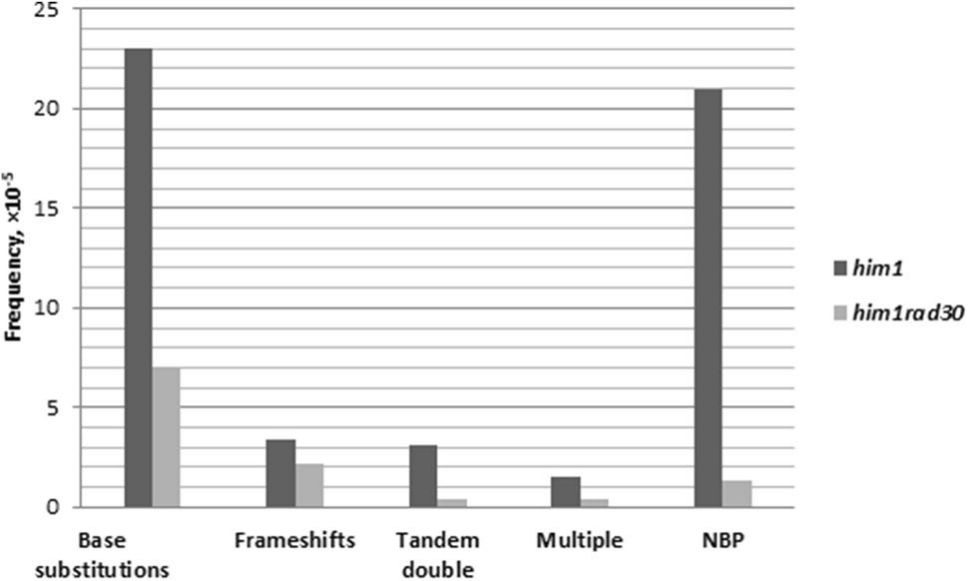
The frequencies and types of base changes associated with defined sites induced by UV-light in him1Δ (1-EAA-3031) (Polη works) and him1Δ rad30Δ (5-EAA-3031) (Polη does not work)

Genetics analysis of the molecular nature of *ade2* mutants has revealed that *him1-1* mutation increases specifically the yield of UV-induced transitions (AT → GC and GC → AT) in comparison with wild-type strains (Ivanov et al. 1987b). We compared the types of base changes induced by UVC in *him1Δ* and *him1Δrad30Δ* strains (Table 3). The comparison of types of base changes observed in *him1Δ* and *him1Δrad30Δ* strains exhibits major differences. The frequency of transitions was robust decreased in *him1Δ rad30Δ* strain in comparison *him1Δ* strain. The frequency of UV-induction mutations localized in the NBP sites (non-bipyrimidine sites) also varied significantly between *him1Δ* (21 × 10^–5^) and *him1Δ rad30Δ* (1.3 × 10^–5^) (Fig. 4)*.* Taken together, data obtained show a significant role of Polη in *him1*-dependent mutagenesis, especially at NBP sites.

**Table 3:** Types of base substitutions induced UV-light

	*him1Δ*		*him1Δ rad30Δ*	
	*n* (%)	*f* × 10^–5^	*n* (%)	*f* × 10^–5^
Transitions				
GCAT	13 (46)	11.5	3 (19)	1.3
ATGC	6 (21)	5.6	3 (19)	1.3
Transversions	9 (33)	8	10 (62)	4
GCTA	1	0.8	1	0.4
ATTA	6	5.6	5	2
TAAT	1	0.8	2	0.8
TAGC	–	–	1	0.4
CGAT	1	0.8	1	0.4
Total	28	–	16	–

What is the reason for the change of polymerases in *him1* mutant? It is known that during the PRR the overwhelming majority of D-loops dissociate without the formation of crossover exchanges (Freidberg et al. 2006). The length of the newly synthesized DNA in the D-loop depends on the rate of DNA synthesis and the probability of termination of this process. As a result of the stochasticity of the termination process, the length of the newly synthesized DNA will be different in each injury bypass event. However, the average length of the synthesized section will depend only on the speed of the synthetic process. The rate of DNA polymerase synthesis depends on the concentration of deoxynucleotides. Therefore, with a decrease in the concentration of dNTP, the average length of the synthesized section will decrease and with a significant decrease in concentration, it will not be able to bridge the gap in the damaged DNA. In *Saccharomyces cerevisiae*, dNTP pools increase by about three-fold upon entry into S-phase relative to G_1_ levels (Szyika et al. 2008). dNTP levels show a three- to five-fold increase in response to DNA damage relative to a normal S-phase, through the check-point-dependent induction of *RNR* genes, the allosteric regulation of RNR activity and the degradation of the Rnr1 inhibitor Sml1 (Zhao et al. 1998, 2001; Chabes et al. 2003). This increase in the amount of dNTP correlates with tolerance to DNA damage.

**Fig. 5:**
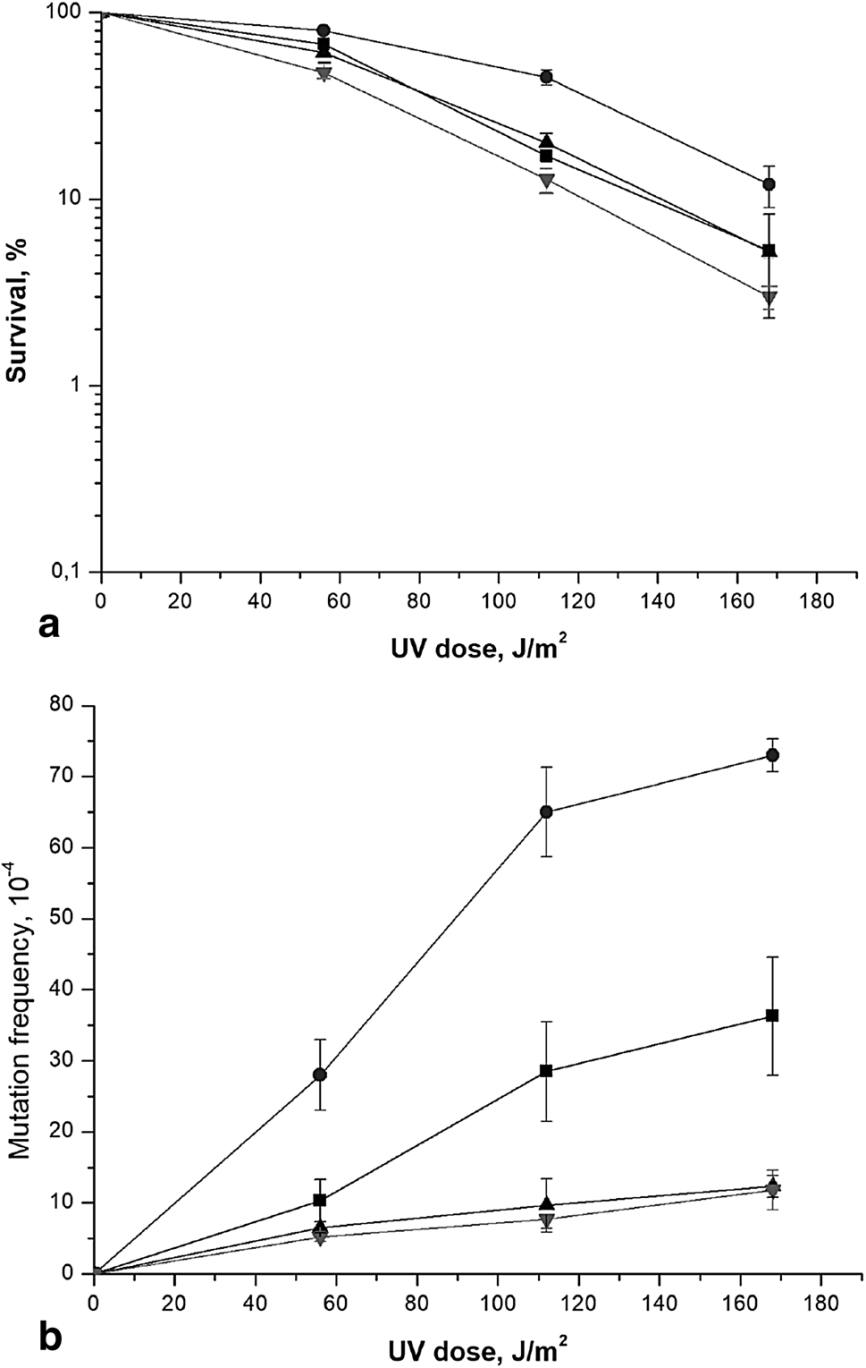
UV-induced sensativity (**a**) and mutagenesis (**b**) in various mutant strains. Isogenic derivatives of strain 11D-3031, were UV-irradiated. The survival and mutation frequencies were determined as described for Fig. 3. The wild type strain (11D-3031) (closed square); *himΔ* (1-EAA-3031) (closed circle); *sml1Δ* (6-DVF-3031) (closed triangle) and* him1Δ sml1Δ* (7-DVF-3031) (closed inverted triangle)

In order to test the effect of the concentration of dNTP on *him1*-dependent mutagenesis, we deleted the *SML1* gene, which encodes a specific suppressor of the *RNR1* gene (*RNR3* homologue), in wild-type and *him1* mutant strains. As a result, the expression of the RNR complex is significantly increased and, as a consequence, the concentration of dNTP also increases by a factor of 2–3 (Zhao, Muller, Rothstein 1998). As can be seen from Fig. 5, deletion of the *SML1* gene lowers the level of UV-induced mutagenesis in comparison with the wild-type strain. Our experiments also demonstrated that *sml1Δ* is able to suppress the higher UV-induced mutagenesis of *him1Δ *mutant (Fig. 5). Thus significant increase in the dNTP levels suppress *him1*-dependent mutagenesis.

**Fig. 6:**
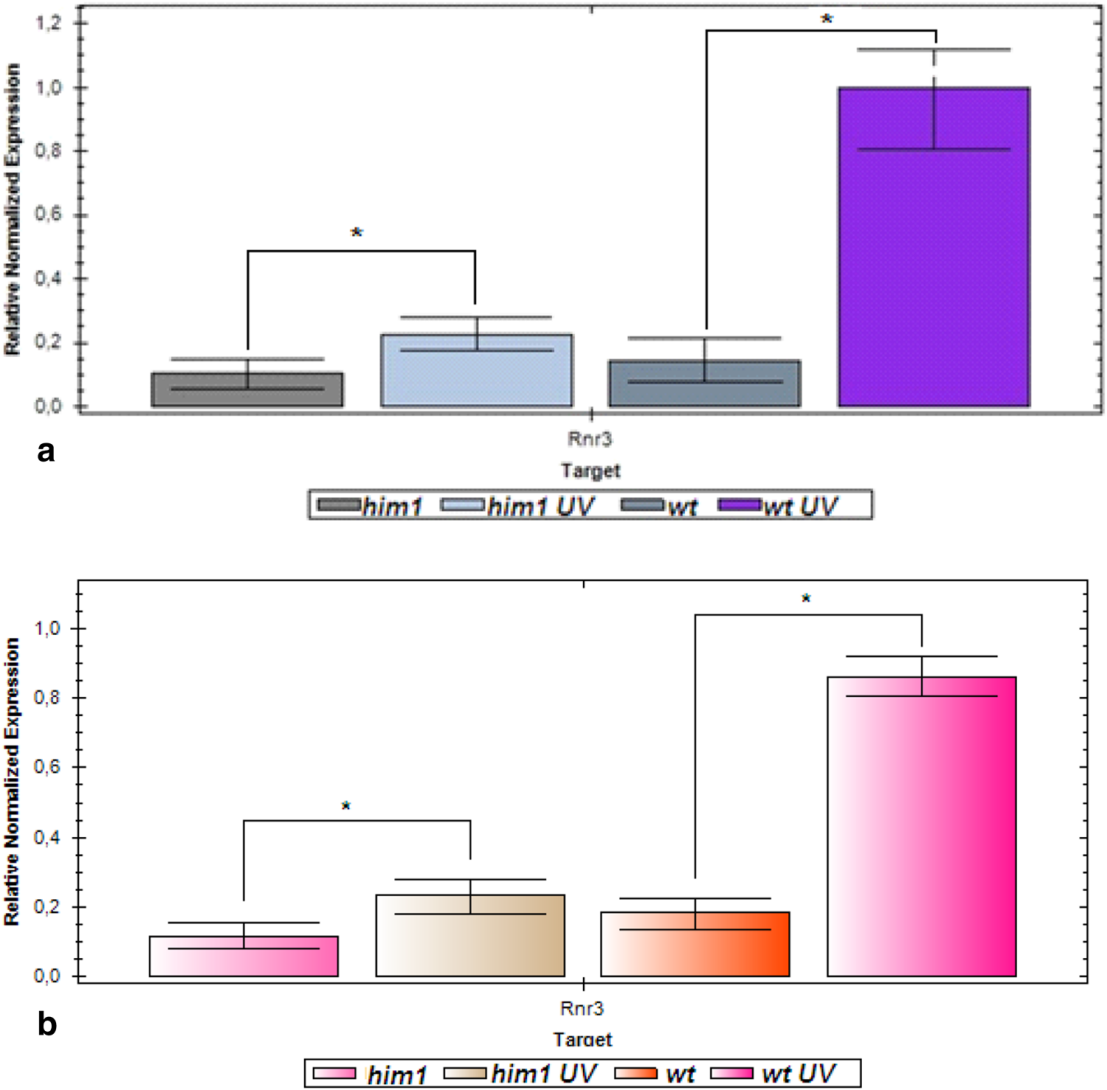
Relative normalized expression of the RNR3 gene in wild-type (11D-3031) and *him1Δ* (1-EAA-3031) mutant cells before and after UV irradiation. **a **UV dose at 252 J/m^2^; **b **UV dose at 140 J/m^2^. *p > 0.05, Student’s *t* test

To test the hypothesis that dNTP concentration regulates the choice of polymerase for loop extending, we examined UV-irradiated *him1Δ* cells for increased *RNR3* expression. We irradiated with UV light the wild-type and *him1Δ* mutant cells and, after 2 h, measured the mRNA *RNR3* gene levels in the irradiated cells. As can be seen from Fig. 6, the mRNA level in wild-type cells increased sevenfold, while in mutant cells the increase did not reach twofold. This finding suggests that *him1Δ* mutation significantly reduces the efficiency of the induction expression of *RNR* genes after UV irradiation. Reduced expression of *RNR* genes will lead to decrease in dNTP concentration. Taken together, the results confirm the hypothesis that intermediate dNTP concentrations stimulate Polη recruitment to fill the gaps. Polη belongs to the highly erroneous polymerase family and this is the cause of the increased UV-induced mutagenesis in *him1Δ* mutant.

## Discussion

The DNA damage response is a powerful intracellular network that has the potential to repair damage and induce cell cycle arrest. To ensure that the DNA damage response works to the benefit of its host cell, it is essential that responses to DNA damage are properly regulated. Here, we have shown that Him1, a protein with unknown function and involved in the regulation of UV-induced mutagenesis and *him1Δ* mutant Polη in *him1Δ* mutant take part in reparative DNA synthesis.

Previous experiments have shown that *him1-1* mutation displays weak mutator phenotype, increasing fivefold the level of spontaneous reversions of the *ade2-42*. Spontaneous mitotic gene conversion in the *ADE2* locus in most heteroallelic combinations is increased (~ twofold) in *him1Δ* strains compared to the wild-type strain (Ivanov et al. 1989). Epistatic analysis showed a synergistic interaction of *him1Δ* with *pms1Δ*, *apn1Δ*, and *rad2Δ* mutations, and *rev3Δ* epistasis with *him1Δ*. Based on the data obtained, we proposed that *HIM1* gene participates in the control of processing of DNA damage that appears after UV irradiation (Kelberg et al. 2005). Here, we have shown that proteins Mms2, Xrs2, involved in the error-free branch of the PRR, have a crucial function in *him1*-dependent UV mutagenesis.

At what stage of the PRR process is *HIM1* taking part? We have previously shown that inactivation of two anti-recombination helicases Srs2 and Mph1 suppresses *him1*-specific mutagenesis (Alekseeva et al. 2018). Both of these helicases destabilize the D-loops and decrease the average length of the synthesized DNA region (Rong and Klein 1993; Dupaigne et al. 2008; Colavito et al. 2009; Panico et al. 2010; Prakash et al. 2009). Consequently, their inactivation will lead to an increase in the length of the newly synthesized DNA in gaps, and this event is the reason for the suppression of *him1*-specific mutagenesis. On the other hand, deletion of the *HSM2* gene (*HMO1*), the product of which stabilizes the D-loops, results in a phenotype similar to *him1Δ* mutation (Ivanov et al. 1989; Alekseev et al. 2002; Kim and Livingston 2006, 2009; Gonzlez-Huici et al. 2014).

From this, it follows that the change of polymerases occurs after the destruction of the D-loop and the gap is filled with an erroneous polymerase.

Earlier, we have shown that *him1-1* and *pms1Δ* mutations have a synergistic effect on UV-induced mutagenesis (Kelberg et al. 2005). The level of induced mutagenesis in *him1Δ pms1Δ* double mutant was significantly higher than in both single mutants. On the other hand, single *pms1Δ* mutant and the wild-type strain have the same level of UV-induced mutations. This is not surprising, since an incorrectly incorporated nucleotide opposite to UV-induced damage (pyrimidine dimers) is not a substrate for mismatch repair. Furthermore, disruption of *PMS1* gene in double *hsm2Δ him1Δ* mutant increases the UV-induced mutation frequency in the triple mutant up to the frequency of single *hsm2Δ* mutant (Kelberg et al. 2005). These data indicate that after the destruction of the D-loop, the gaps are filled with an erroneous Polη. Since the mismatch repair *PMS1* gene is not directly related to damage-induced mutagenesis, we suggested that mismatch repair substrates arose in the cells of *him1Δ* mutant as a result of the attraction of this polymerase to the synthesis of DNA into the gaps. Consistent with this conclusion, we found that Polη inactivation completely blocks *him1*-dependent UV mutagenesis. On the other hand, our data showed that Polη-dependent mutagenesis in NBP sites occurs significantly more frequently in *him1Δ* mutant than in the double *him1Δ rad30Δ* mutant. This result indicates that during the DDT, the Polη effectively works on an intact template into the gaps.

**Fig. 7:**
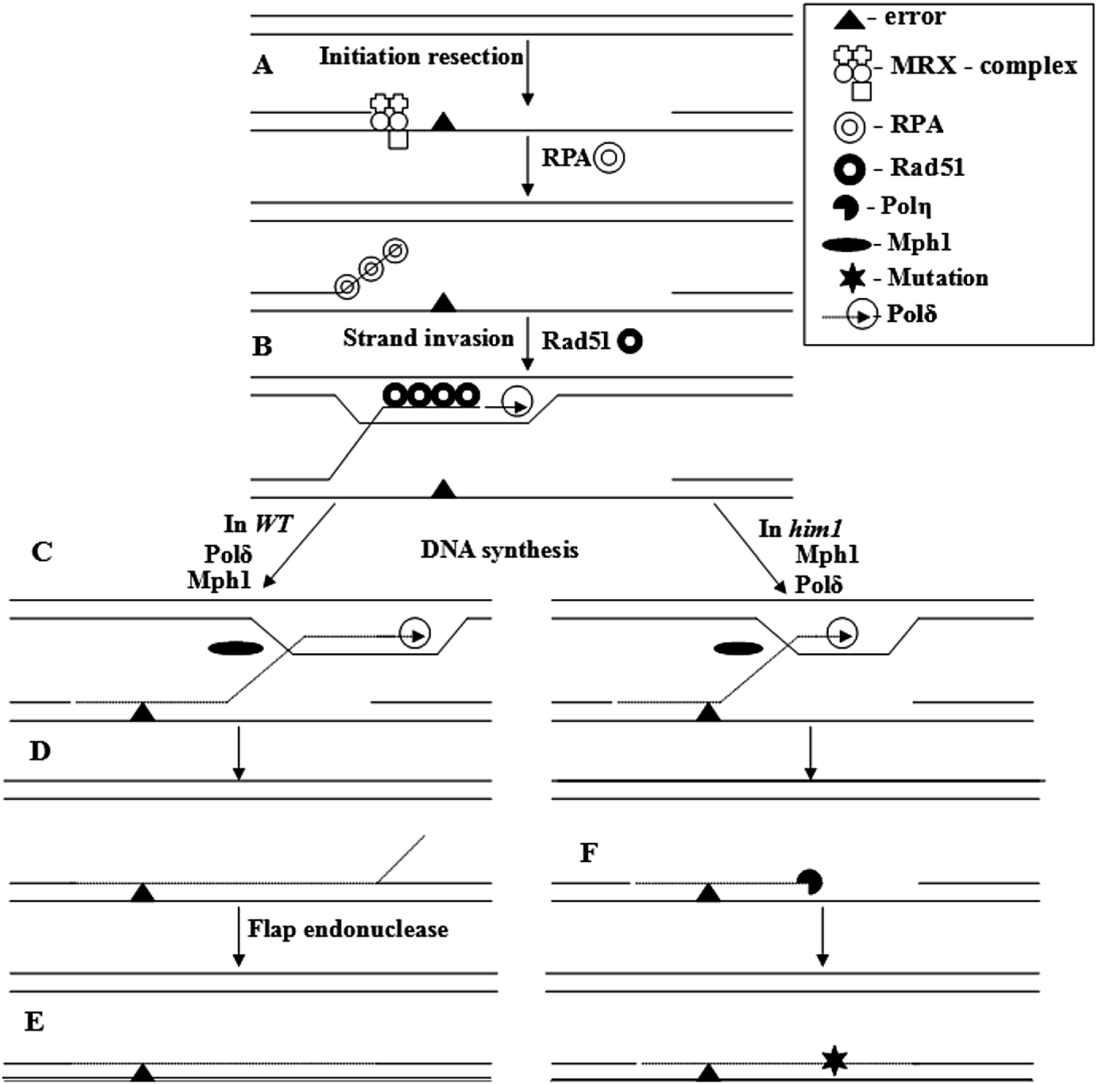
The process of DNA damage error-free bypass. Stall replicative complex is recognized by the Mre11-Rad50-Xrs2 (MRX) complex. MRX initiates resection of newly synthesised DNA strand. The exposed ssDNA is bound by RPA. After RPA is replaced by Rad51, the Rad51 filament is formed, which is introduced into the sister chromatid, leading to the formation of D-loop. Details in the text

On the basis of the data obtained, it is possible to construct a hypothetical mechanism for an error-free repair branch (Fig. 7). After the competition for access to the primer end of DNA in the stopped replication fork wins the MRX complex. The degradation of the newly synthesized DNA strand begins and a gap is created before the damage (Fig. 7a). The released 3′- end is coated with RPA protein, which attracts recombination machinery (Rad51, Rad52, Rad55, Rad57) and a nucleoprotein filament is formed (Fig. 7b). The specific helicase Srs2 destroys a significant portion of these filaments, displacing the Rad51 protein. The rest of the filaments are introduced into sister chromatid, forming a D-loop structure (Fig. 7c). Rad51 protein-free filaments are possibly captured by the Rad5 protein and are also incorporated into sister chromatid. As a result, D-loop structures are generated. At the same time, another specific helicase Mph1 and other proteins, begin, the effective work of which ultimately leads to the destruction of the hybrid DNA (Fig. 7d). The length of the newly synthesized DNA region will depend on the ratio of the speeds of the synthesis of the strand and hybrid DNA destruction. Normally, in wild-type cells, the rate of synthesis due to the extremely high concentration of deoxynucleotides apparently will be approximately equal to the rate of unwinding of the strands. As a result, the length of the newly synthesized fragment of the strand will exceed the length of both gaps around the damage and an error-free damage bypass will take place (Fig. 7e).

We proposed and showed that mutations in *HIM1* gene affect the efficiency of the induction of the RNR complex and, as a result, decrease the concentration of deoxynucleotides after UV in the nucleoplasm. This leads to a drop in the rate of repair DNA synthesis and shifts the equilibrium towards the premature collapse of the hybrid DNA. After annealing the released strand on the mother duplex, the 3′-terminus occupies Polη (Fig. 7f). After the first error, Polη will be replaced by Polζ, a good substrate for which are unpaired bases at the end of the primer. The latter, apparently, will complete the development of the remaining gap.

In conclusion, this study identifies the *HIM1* gene as a novel member of error-free pathway of DDT (Figs. 1, 2). Interestingly, the mechanism through *him1Δ* mutant acts by recruiting polymerase Polη to carry out reparative DNA synthesis during DDT. It will be interesting in the future to determine how does the Him1 protein affect the regulation of RNR gene expression.
